# Infection of juvenile falcons (*Falco* spp.) with intestinal *Lawsonia intracellularis*

**DOI:** 10.1002/vms3.1063

**Published:** 2023-01-14

**Authors:** Peter Wencel, Sionagh H Smith, Liesbeth Couck, Tom Hellebuyck, Peter C Scott, Steven McOrist

**Affiliations:** ^1^ Al Aseefa Falcon Hospital Dubai United Arab Emirates; ^2^ Royal (Dick) School of Veterinary Medicine University of Edinburgh Edinburgh UK; ^3^ Faculty of Veterinary Medicine Ghent University Merelbeke Belgium; ^4^ Scolexia Avian and Animal Consultancy Co. Moonee Ponds Victoria Australia

**Keywords:** avian, falcon, Falconiformes, PCR, *Lawsonia intracellularis*

## Abstract

Intestinal infection of many host species with *Lawsonia intracellularis* are widely reported. Analyses of infections among carnivorous falcons have not previously been reported. Fifty juvenile captive falcons (*Falco* spp.) with or without *Lawsonia* infection were investigated in the United Arab Emirates, including clinical laboratory methods. Fresh intestinal biopsy samples were analysed by microbiological techniques for *Lawsonia* and other bacteria and by standard parasitological and pathological methods. *Lawsonia intracellularis* infection was diagnosed by microbiological examination and qPCR in 10 of 50 juvenile falcons at case examination. Seven of these 10 falcons were of normal clinical appearance, and the other three had other contributing factors to ill‐thrift. A range of other conditions were noted in 40 case control falcons. This first report of *Lawsonia* infection in falcons suggests that the agent may have a limited contribution to clinical disease in these birds, including ill‐thrift syndromes. This lack of clinical disease association mimics that noted among *Lawsonia* infections recorded in other avian families.

## 1 INTRODUCTION

Birds in the order Falconidae (falcons) have narrow pointed wings, keen eyesight, and a tomial tooth beak formation. Falcons are carnivorous and chase small live prey, such as rodents, and kill them using their beaks (Mindell et al., 2018). Among the 40 falcon species, peregrine falcons (*Falco peregrinus*), saker falcons (*Falco cherrug*), gyrfalcons (*Falco rusticolus*), and their various hybrids are sometimes raised and conditioned in captivity for exhibition or sporting purposes (Fleming et al., 2011). Falcons bred and raised in captivity are typically fed small rabbits or rodents, such as mice, hamsters, or rats and/or small birds such as young quail, ducks, or chickens (Fleming et al., 2011). Juvenile and adult falcons can suffer a range of clinical conditions, such as ill‐thrift and diarrhoea, due to campylobacteriosis, mycobacteriosis, dietary mis‐management, or coccidiosis (*Caryospora* sp.) (Kubiak & Forbes, 2011; Samour, 2006).

*Lawsonia intracellularis* is an obligate intracellular bacterium identified as the causative agent in intestinal lesions of proliferative enteropathy in a wide range of animal species (McOrist & Gebhart, 2005). In some mammalian hosts (pig, horse, hamster, rabbit), the infection is both commonly reported and an important cause of clinical ill‐thrift syndromes, whereas in many other hosts, it appears to be a relatively rare and unusual clinical event. *Lawsonia intracellularis* is a single‐strain bacterium; its entry mechanisms and intracytoplasmic life cycle within intestinal epithelial cells appear to be identical in all host species investigated so far (McOrist et al., 1989). Infection of immature intestinal crypt cells by *L. intracellularis* is consistently associated with proliferation of these cells, due to *Lawsonia*‐mediated failure of differentiation into mature villous cells, with a consequent reduction in intestinal absorptive capacity (McOrist et al., 1989).

The purpose of this study was to investigate juvenile falcons presented for veterinary attention, including ill‐thrift, at avian medicine clinics in the United Arab Emirates.

## 2 MATERIALS AND METHODS

Besides local wild populations, the captive falcon breeding facilities in the United Arab Emirates consist of approximately 1000 breeding pairs of various falcon species (*Falco* spp.) and their crosses. Several thousand juvenile falcons (less than 1 year old) are also transported annually into the United Arab Emirates from breeding facilities located in Europe and North America. Avian clinics in the United Arab Emirates are therefore responsible for monitoring the health of approximately 1000 juvenile and 2000 adult falcons each year, with each bird considered of high cultural and economic value.

We performed case investigations of 50 juvenile falcons presented to two avian clinics (in 2019–2020) for the evaluation of health status, with both healthy birds and other presentations including poor appetite or failure to achieve target sporting performance. The case investigations included clinical history, clinical examination, whole body radiographs, endoscopy with bacteriological and parasitological examination of faecal and crop swab samples, along with blood haematology and biochemistry samples, each collected and processed by routine clinical laboratory methods and then compared to known reference ranges.

Further investigations of selected cases enrolled in this study included the following: modified acid‐fast, Romanowsky and Gram staining of separate smears of lower intestinal tract biopsy samples for *L. intracellularis*, conducted using methods described previously (Love et al., 1977), semi‐quantitative PCR specific of these samples (100 mg per bird) for *L. intracellularis*, conducted using preparation methods and oligo‐primers as described previously (Nathues et al., 2009). Among pathology investigations, lower intestinal tract biopsy samples from three birds (# 1, 6 and 7; selected opportunely) were routinely processed for histopathology, sectioned at 3 μm and stained by haematoxylin and eosin, or Warthin–Starry silver impregnation. Cohort biopsy samples from these birds were also fixed in 1% glutaraldehyde in 0.1 M sodium cacodylate buffer. After routine processing and staining with uranyl acetate/lead citrate, ultra‐thin sections were examined via transmission electron microscopy.

## 3 RESULTS

The results of case investigations are summarized in Table 1. The falcon species presented to avian clinics in the United Arab Emirates for this study: gyr falcon (*F. rusticolis*), peregrine falcon (*F. peregrinus*) or cross breed (gyr/peregrine) were considered typical of falcons held in local captive bird facilities. In 10 juvenile falcons (# 1 to # 10), bacterial examination and qPCR results were consistent with intestinal *L. intracellularis* infection (see Table 1). Seven of these 10 falcons were presented for veterinary examination in the absence of clinical signs, and only three were presented for investigation of clinical ill‐thrift. Other identified causes of ill‐thrift and/or poor performance in the latter three falcons and in un‐infected case controls included husbandry mis‐management, aspergillosis, bacterial (*Campylobacter* spp.), or coccidial (*Caryospora* spp.) enteritis (see Table 1). Segmented filamentous bacterial forms were visualized in association with the intestinal epithelium in 10 cases (data not shown) and were considered commensal bacteria (Hedblom et al., 2018).

**TABLE 1:** Selected features of investigations of juvenile falcons presented to veterinary clinics in the United Arab Emirates

Case No.	Falcon species	Clinical presentation	Faecal Campylobacter culture	Faecal endoparasite examination	Gut biopsy intracellular bacterial stain	Gut biopsy *Lawsonia* qPCR assay	Pathology findings
1	Gyr × Peregrine	Healthy	^−^	^−^	^+^	10^3.9^	Intracellular vibrioid bacteria
2	Peregrine	Healthy	^−^	*Capillaria* sp.	^+^	10^3.2^	Candidiasis
3	Gyr	Ill‐thrift	^+^	^−^	^+^	10^4.2^	ND
4	Gyr	Healthy	^−^	^−^	^+^	10^0.1^	ND
5	Gyr × Peregrine	Healthy	^−^	^−^	^+^	10^3.5^	ND
6	Peregrine	Healthy	^+^	^−^	^+^	10^1^	No significant findings
7	Gyr × Peregrine	Ill‐thrift	^−^	^−^	^+^	10^1.5^	Aspergillosis
8	Gyr	Healthy	^−^	*Caryospora* sp.	^+^	10^1^	ND
9	Gyr × Peregrine	Ill‐thrift	^+^	−	^+^	10^4.7^	Diffuse pneumonia
10	Gyr	Healthy	^−^	*Caryospora* sp.	^+^	10^3^	ND
11–25	Gyr	Healthy	^−^	^−^	^−^	^−^	ND
25–40	Gyr × Peregrine	Healthy	^−^	^−^	^−^	^−^	ND
40–50	Gyr	Ill‐thrift	^−^	*Caryospora* sp.	^−^	^−^	ND

*Note*: qPCR assay results expressed as log *Lawsonia* bacteria per mg of sample (Nathues et al., 2009).

Abbreviation: ND, not done.

^−^ Negative test result.

^+^ Positive test result.

**FIGURE 1:**
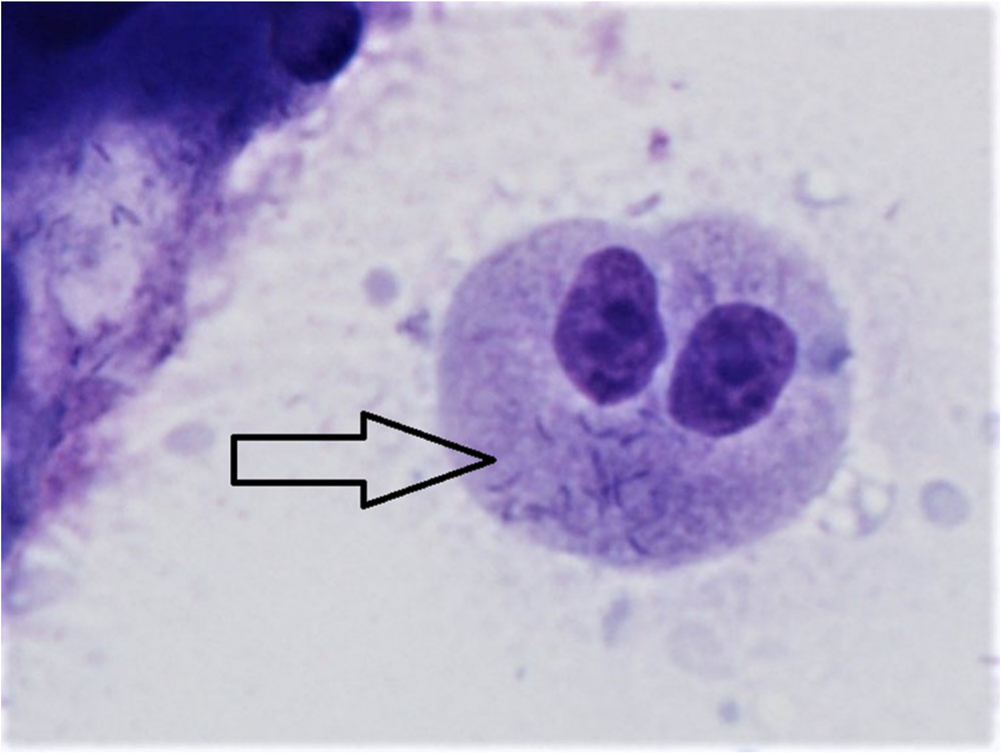
Intestinal biopsy of falcon intestinal epithelial cells. Numerous intracytoplasmic curved bacteria (arrow) in immature epithelial cell. Romanowsky stain.

Staining of lower intestinal tract biopsy samples in cases # 1 to # 10 typically showed curved, intracellular Gram‐negative bacteria within immature intestinal epithelial cells (see Figure 1). Identical staining of samples from other cases was consistently negative for such bacteria.

**FIGURE 2:**
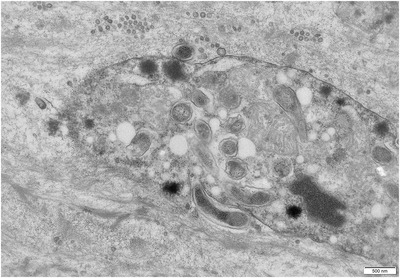
Intestinal biopsy of falcon intestinal epithelial cells. Numerous intracytoplasmic vibrioid bacteria in immature epithelial cell. Uranyl acetate/lead citrate stain.

Transmission electron microscopy of biopsy samples from selected cases with bacterial examination and qPCR evidence of *L. intracellularis* infection confirmed immature intestinal epithelial cells containing intracytoplasmic bacteria. These bacteria were curved, vibrioid, and Gram‐negative (tri‐laminar envelope), measuring 1.25–1.75 μm long and 0.25–0.4 μm wide (see Figure 2). The organisms were consistent with *Lawsonia* phenotype, consistently located free in the cytoplasm and occasionally noted in apposition to cell mitochondria (see Figure 2). Histopathology examination of limited cohort biopsy samples from these selected cases indicated normal crypt/villus architecture, with no distinct intracellular elements.

## 4 DISCUSSION

Infection of immature intestinal cells with *L. intracellularis* has been reported in numerous animal groups, and we report it here for the first time in falcons (*Falco* spp.). In previous surveys of ill‐thrift and bacterial infections of falcons, *Lawsonia* has not been detected (Bangert et al., 1988; Kubiak & Forbes, 2011; Samour, 2006). While the infection appeared to be not uncommon in falcons in this study (10 of 50 case investigations), its level of pathogenicity to this host genus is not clear. In most cases, we found that the infection was apparently subclinical and self‐limiting, with no long‐lasting ill‐effects, despite case investigations aimed at identifying causation of ill‐thrift or poor performance. Our case investigations also confirmed the occasional presence of other known causes of ill‐thrift among juvenile falcons, such as coccidiosis, fungal infection, and bacterial enteritis.

*Lawsonia intracellularis* forms a homogenous genus and species within the Desulfovibrio family, with no genetic differences between isolates collected from different animal hosts (Bengtsson et al., 2020). This lack of speciation indicates that the bacterium is of recent evolutionary lineage (Schmitz‐Esser et al., 2008). *Lawsonia* requires intracellular mitochondrial sources of energy and appears to be capable of an induced phagocytosis towards intestinal epithelial cells of many avian and mammalian hosts (Schmitz‐Esser et al., 2008). This process was also evident in the infected falcons in this study (see Figure 2).

*Lawsonia* is considered capable of interrupting normal crypt cell differentiation into mature villus cells, thereby causing the distinctive lesions of proliferative enteropathy noted in many host animal species (McOrist et al., 1996). While in some hosts (pigs, horses, hamsters, rabbits), *Lawsonia* infections are clearly associated with significant lesions of proliferative enteropathy, in many other groups of animals, it occurs as rare and irregular recorded events (e.g., dogs, rats, foxes, monkeys) (Cooper & Gebhart, 1998). However, in avian hosts, members of both the Passeriformes and Galliformes appear to be relatively resistant to *Lawsonia* infection, with very low infection rates and repeated failure to demonstrate susceptibility upon challenge exposure (Collins et al., 1999; McOrist et al., 2003; Ohta et al., 2017; Viott et al., 2013). It is naturally likely that juvenile falcons have a higher rate of exposure to *Lawsonia* due to the carnivorous consumption of rodents or rabbits in their diet. While advisable, these potential sources of infection are not routinely tested. It is also possible that, in common with the other aforementioned bird groups, the gastrointestinal tract of falcons may become infected, but without sustained infection or significant disruption to intestinal architecture.

## AUTHOR CONTRIBUTIONS

All authors contributed to study design, execution, and reporting.

## CONFLICT OF INTEREST

The authors declare no conflict of interest.

## FUNDING INFORMATION

No financial support was received from any institution for this research.

### ETHICS STATEMENT

The live animal research conducted in the study took place with the approval of the animal ethics oversight body of the United Arab Emirates.

### PEER REVIEW

The peer review history for this article is available at https://publons.com/publon/10.1002/vms3.1063.

## Data Availability

Data available on request from authors.
